# Supplementary material for: Early N-Terminal Pro B-Type Natriuretic Peptide (NTproBNP) Plasma Values and Associations with Patent Ductus Arteriosus Closure and Treatment—An Echocardiography Study of Extremely Preterm Infants
Source: J Clin Med. 2022 Jan 27;11(3):667. doi: 10.3390/jcm11030667 (PMC8837127; doi:10.3390/jcm11030667)
Supplement: Supplementary file 1 [file jcm-11-00667-s001.zip › jcm-1514019-supplementary.pdf]

Supplemental Table S1. Clinical local guidelines for diagnosing and treating a haemodynamic significant patent ductus arteriosus (hsPDA) at the Karolinska University Hospital during 2012-2014*		
	Echocardiographic criteria of a hsPDA	Echocardiographic projection details
**Ductal characteristics**		
Diameter	Diameter of the duct >1.5mm	Ductal diameter was acquired with two-dimensional mode at the narrowest diameter of the ductal pulmonal end, in a high parasternal short axis (ductal cut)
Direction of flow through the duct	Left to right shunt (L->R)	Colour Doppler was used to detect the PDA and the direction of the flow (left to right, bidirectional or right to left)
Flow characteristics	Pulsatile flow through the duct with a non-restrictive flow profile	Duct visualized from ductal cut. Velocity through duct was measured either with pulsed wave Doppler or continuous wave Doppler
**Excessive pulmonary flow**		
Left atrium	LA:Ao ratio >1.5	Left atrial diameter and aortic root dimension was determined in two-dimensional mode according to guidelines and standards in a parasternal long axis view
Pulmonary flow characteristics	End-diastolic flow in left or right pulmonary artery >0.2m/s	Diastolic forward flow velocity measured with pulsed wave Doppler in left or right pulmonary artery from a high parasternal short axis view
**End-organ ductal steal**		

	Signs of ductal steal (reversed diastolic flow in descending aorta /reversed or absent flow in mesenteric artery and/or anterior cerebral artery	Pulsed wave Doppler was used to detect reversed diastolic flow in descending thoracic aorta and images were aquired from a high parasternal view. The subcostal view was used for abdominal aorta or mesenteric reversed or absent diastolic flow. Pulsed wave Doppler was used to assess reversed diastolic flow in abdominal aorta, reversed or absent diastolic flow in mesenteric artery and/or anterior cerebral artery.
**Heart failure and ventricular enlargement**		
	Visual signs of left ventricular enlargement. Assessment of left ventricle end-diastolic diameter (compared with reference values).	Left ventricle diameter was determined in two-dimensional mode according to guidelines and standards in a parasternal long axis view
Abbreviations: hsPDA: haemodynamic significant patent ductus arteriosus; LA:Ao: left atrium: aortic root ratio<br>*The treatment decision was at the discretion of the attending neonatologist. Clinical signs such as respiratory status with invasive ventilation and fraction of inspired oxygen, feeding intolerance, clinical signs of heart failure and haemodynamic compromise, were combined with the echocardiography findings described in this table.		

Supplemental Table S2. Characteristics of the infants not treated for PDA

	No PDA treatment			
	All	No closure[a,b]	Spontaneous closure[a] at ≤7 days of age	Spontaneous closure[a] at >7 days of age
N=98	N=40	N=7	N=6	N=27
Any antenatal steroids, n (%)	39 (98)	7 (100)	6 (100)	26 (93)
Multiple birth, n (%)	10 (25)	2 (28)	1 (17)	7 (26)
Vaginal delivery, n (%)	10 (25)	3 (43)	1 (17)	6 (22)
Chorioamnionitis, n (%)	17 (43)	4 (57)	3 (50)	10 (37)

Gestational age, weeks, mean (SD)	26.4 (1.1)	24.7 (1.1)	26.4 (0.9)	26.8 (0.9)
Birth weight, grams, mean (SD)	816 (173)	788 (174)	752 (88)	851 (176)
SDS birth weight, mean (SD)	-1.4 (1.4)	-1.0 (1.1)	-2.2 (0.9)	-0.85 (1.0)
Female sex, n (%)	21 (53)	2 (33)	2 (33)	17 (61)
Apgar <7 at 5 min, n (%)	15 (38)	6 (86)	1 (17)	8 (30)
Surfactant within two hours after birth, n (%)	16 (38)	6 (86)	1 (17)	9 (33)
Median age at PDA closure[a], days (IQR)	21 (12-45)	NA	3 (2-3)	28 (18-65)
Death before ductal closure, n (%)	6 (15)	6 (86)	0 (0)	0 (0)
[a] date of ultrasound confirmed ductal closure or date of surgery				

[b] infant transferred before closure so date only known in 33/40 infants as six died before closure

Abbreviations: PDA: patent ductus arteriosus; SD: standard deviation; IQR: interquartile range; NA: not applicable

Supplemental Table S3. Censoring protocol for the Kaplan Meier survival curves of ductal closure in the cohort

Outcome	Number of infants	Censoring	Comment
Spontaneous closure without PDA treatment	32	On date of confirmed ductal closure with ultrasound	One infant died after spontaneous closure, but had already been censored
Closure after ibuprofen PDA treatment	25	On date of confirmed ductal closure with ultrasound	
Closure after PDA surgery	5	On date of PDA surgery	

Closure after ibuprofen and later PDA surgery	17	On date of PDA surgery	
Death without PDA treatment	6	On date of death	
Death after PDA treatment	5	On date of death	
Transfer to another Swedish hospital out of Stockholm region before closure, without PDA treatment	1	On date of transfer	
Transfer to another Swedish hospital out of Stockholm region	4	On date of transfer	

before closure, after PDA treatment			
Closure after PDA treatment at 40 weeks postmenstrual age*	2	At assigned ductal closure at 40 weeks as no later ultrasound available	
Spontaneous closure at 40 weeks postmenstrual age*	1	At assigned ductal closure at 40 weeks as no later ultrasound available	
*Three infants were assigned a closure date at full-term age			

Supplemental Table S4. Comparison of the PDA closure age as estimated by medians and by 50% closure with Kaplan-Meier survival analysis estimates

	Median closure age in days (IQR)†	Age in days at 50% closure rate (25%; 75% rate)††
All infants in the cohort	N=82<br>23 (15-57)	N=98<br>45 (20;83)
By gestational age		
- At less than 25 weeks	N=14<br>18.5 (15-32)	N=24<br>51 (38;57)
- At 25 weeks or higher	N=68<br>24 (14-63)	N=74<br>42 (17;83)
*By ibuprofen doses**		
No ibuprofen treatment	N=33	N=40

	21 (11-46)	24 (13;58)
Ibuprofen doses 3 or less	N=16<br>21 (13.5-47)	N=17<br>55 (15;missing)‡
More than 3 doses ibuprofen	N=28<br>35 (18.5-63)	N=36<br>63 (45;115)
*No treatment or any ibuprofen treatment***		
No treatment	N=33<br>21 (11-46)	N=33<br>21 (11;46)
Ibuprofen only	N=27<br>51 (17-106)	N=27<br>51 (17;106)

†Infants who were transferred or died before ductal closure excluded

††all infants included or as specified in each analysis with an observation time until 133 days of age. From the Kaplan-Meier survival curve, the 50% closure rates with 25% and 75% rates are shown. The analysis does not show the 75% rates in some subgroups due to a limited number of infants

*Infants (N=5) having primary PDA surgery excluded

**Infants later dying, undergoing PDA surgery or being transferred before ductal closure excluded

Supplemental Figure S1a

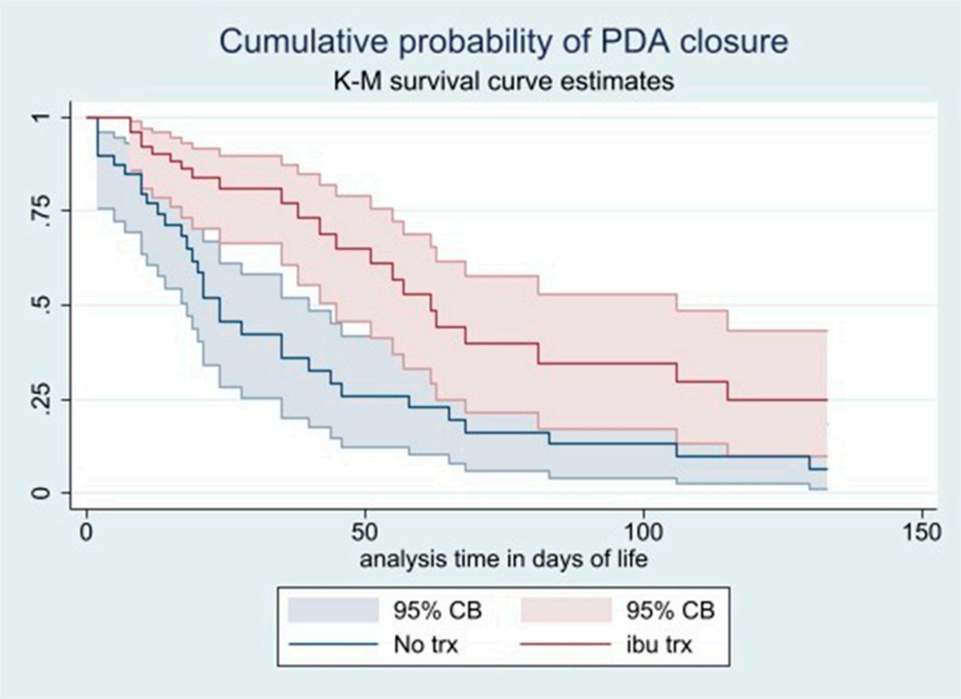

Supplemental Figure S1a. The cumulative probability of PDA closure for all infants, except those who underwent primary PDA surgery (N=93), is shown in a K-M survival curve with 95% CB. The analysis time was until 133 days of age as then all the infants had reached 40 weeks postmenstrual age. The censoring protocol is shown in Supplemental table 3. Infants are divided into those receiving ibuprofen treatment and those not receiving any treatment. Abbreviations: PDA: patent ductus arteriosus; K-M: Kaplan-Meier; CB: confidence bands

Supplemental Figure S1b

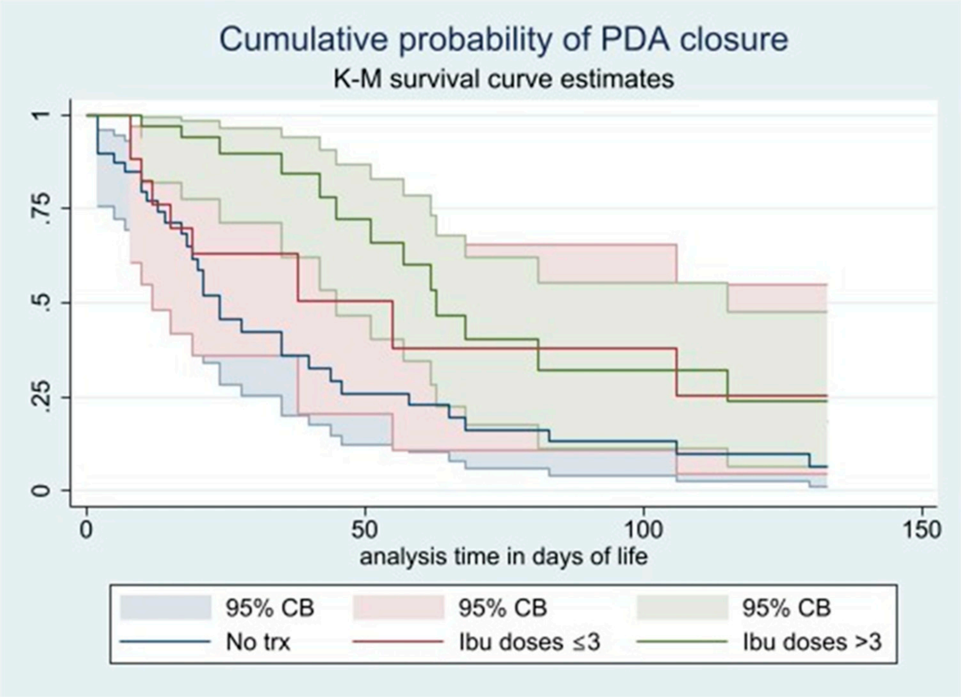

Supplemental Figure S1b. The cumulative probability of PDA closure for all infants except those who underwent primary PDA surgery (N=93) is shown in a K-M survival curve with 95% CB. The analysis time was until 133 days of age as then all the infants had reached 40 weeks postmenstrual age. The censoring protocol is shown in Supplemental table 3. Infants are divided into following categories: no treatment, three doses of ibuprofen or less and more than three doses of ibuprofen. Abbreviations: PDA: patent ductus arteriosus; K-M: Kaplan-Meier; trx:treatment; Ibu: ibuprofen; CB: confidence bands

Supplemental Figure S2

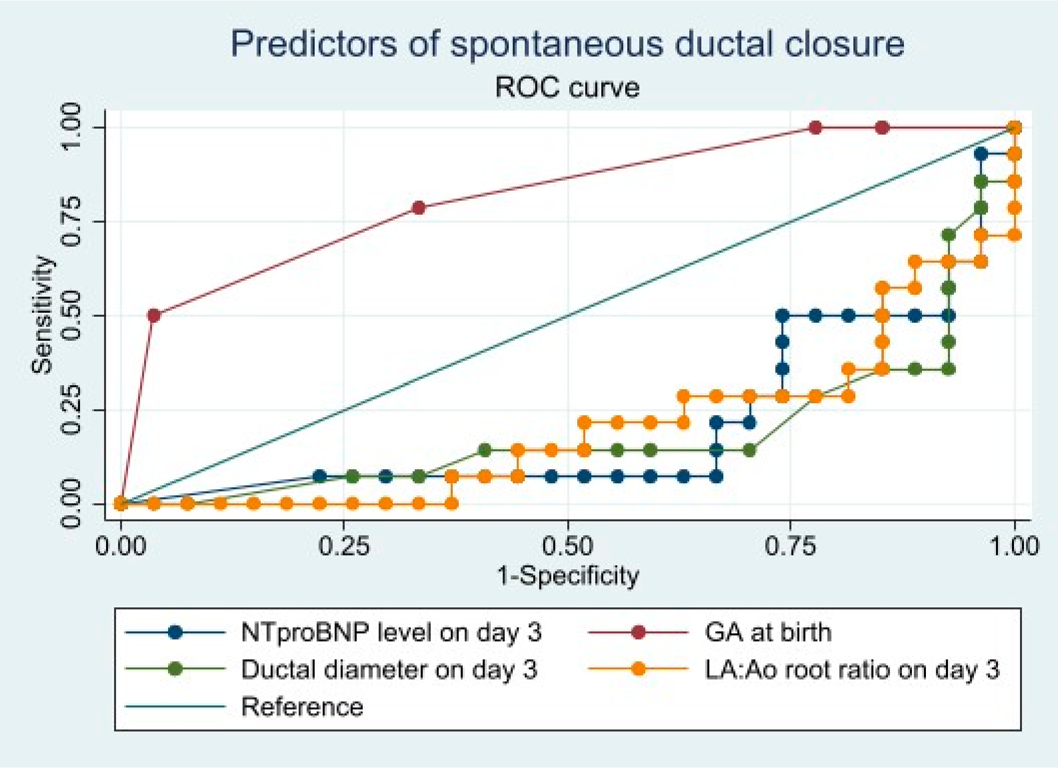

Supplemental Figure S2. The ROC curve is shown for all predictors of spontaneous ductal closure. It was studied how NTproBNP level on day three, GA at birth, diameter of the duct and LA:Ao ratio on day three predicted spontaneous closure. NTproBNP level on day three resulted in an AUC value of 0.31 (95% CI 0.19-0.43), with a sensitivity and specificity of 61% and 20% respectively, for the cut-off value at 6001-9000 ng/L. For GA the AUC was 0.76 (0.67-0.86) with a sensitivity and specificity of 74% and 63% respectively, at a cut-off GA at 26 weeks, in predicting spontaneous ductal closure. The AUC for ductal diameter was 0.18 (0.04-0.33) and LA:Ao ratio the AUC was 0.20 (0.06-0.34).

Abbreviations: ROC: receiver operating characteristics; NTproBNP: N-terminal pro B-type natriuretic peptide; LA:Ao ratio: Left atrium to aortic root ratio; AUC: area under the curve.

Supplemental Figure S3

Predicting spontaneous closure

	Obs	ROC/AUC	Std. Err.	[95% Conf. Interval]	
GA	41	0,8188	0,0672	0,6871	0,9505
NTproBNP-Day 3	41	0,2090	0,0780	0,0562	0,3618
LA:Ao-Day 3	41	0,2011	0,0726	0,0589	0,3433
Ductal diameter-Day 3	41	0,1878	0,0771	0,0366	0,3390

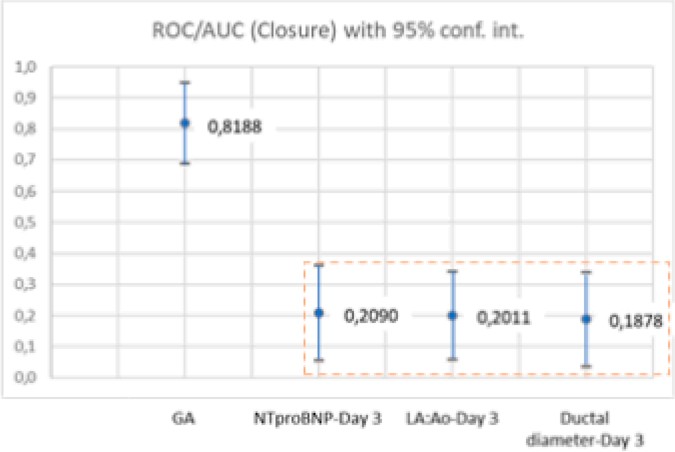

Predicting PDA surgery

	Obs	ROC/AUC	Std. Err.	[95% Conf. Interval]	
NTproBNP-Day 3	41	0,7902	0,0940	0,6060	0,9744
LA:Ao-Day 3	41	0,6897	0,0880	0,5172	0,8621
Ductal diameter-Day 3	41	0,5388	0,0987	0,3453	0,7323
GA	41	0,2126	0,0690	0,0773	0,3480

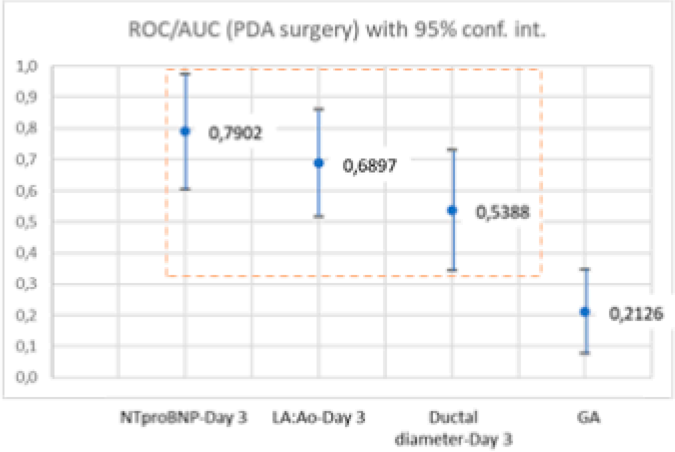

As the comparison of ROC curves shows (STATAcommand roccomp), GA is a notably stronger predictor of PDA closure than NTproBNP-Day 3, LA:Ao-Day 3 or Ductal diameter-Day 3. However, when it comes to predicting PDA surgery it is the NTproBNP-Day 3 as well as LA:Ao-Day 3 and Ductal diameter-Day 3 that provide a considerably better prediction than GA. Thus, the addition of NTproBNP-Day 3 to the overall prediction model with regard to PDA surgery (as one of the outcomes examined in this study) is beneficial and adds to the predictive power. Included in this analysis were all infants who had available NTproBNP, ductal diameter and LA:Ao ratio values on day three (no missing values), N=41.

Comparison of the confidence intervals for the AUC values of the different predictors of spontaneous PDA closure and PDA surgery respectively.

Supplemental Figure S4

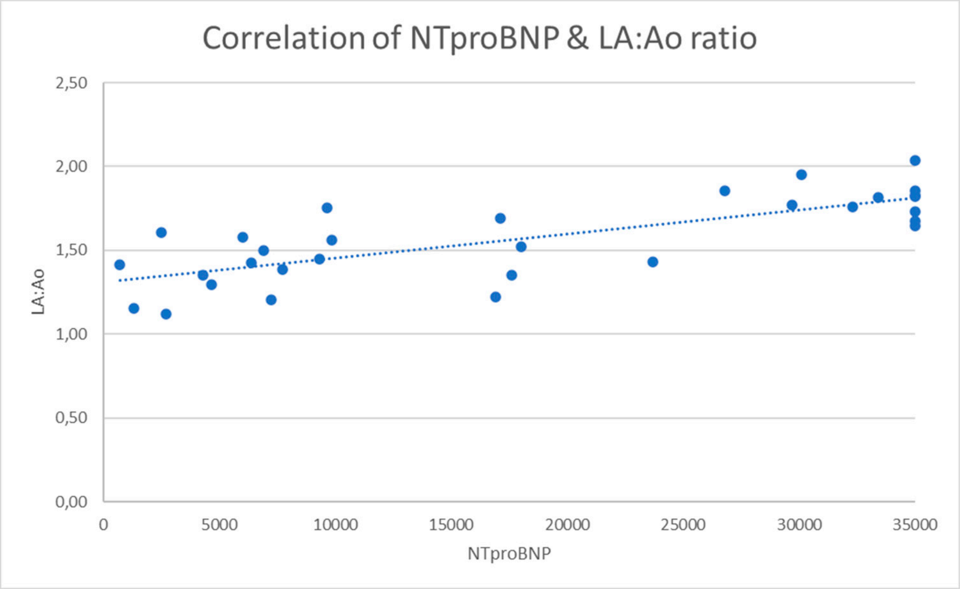

Supplemental Figure S4. The scatterplot shows the relationship between NTproBNP and LA:Ao ratio (N=31). The linear model was used to fit the regression line (R^2 equals to 0.58). The Pearson correlation coefficient is 0.76. As we see, there is a reasonable correlation between these two variables. The presence of correlation in this case does not imply a causation and infers rather to an association between the studied variables.

Abbreviations: NTproBNP: N-terminal pro B-type natriuretic peptide; LA:Ao: Left atrium to aortic root ratio.
